# Alcoholism as a Disease

**Published:** 1880-04

**Authors:** S. F. Bennett

**Affiliations:** Richmond, Ill.


### ARTICLE III.

### ALCOHOLISM AS A DISEASE. By S. F. BENNETT, M.D.

Dr. C. W. Earle's article on "The Cinchona Cure for Intemperance," published in the February number of THE JOURNAL AND EXAMINER, excites my surprise in this—that any one having experience in the use of the drug cinchona rubra, should pronounce its effects nil in the treatment of alcoholism. Of the "cinchona cure" dispensed by the specialist Dr. Earle mentions, I have nothing to say, but I am impressed with the belief that the analogies between that class of diseases in which cinchona is so near a specific, and alcoholism are so remarkable as to make it probable that the same remedy should prove efficacious in both.

For a long time my attention has been called to the curious, and sometimes startling, analogies in the phenomena of alcoholism and so-called "malarial affections." Both have a special causation, the first alcohol, the second a morbific agent commonly known as malaria. The causative relation of alcohol to drunkenness, none will dispute, and it is not germane to discuss here the arguments in favor of the existence of a special morbific agent as the cause of intermittent, remittent and pernicious fevers since it is with the phenomena only of these diseases, and alcoholism, that I propose to deal.

The most singular characteristic of the simplest form of malarial disease, intermittent fever, is periodicity. We have a cold stage, a stage of pyrexia and a sweating stage with intermissions of remarkably equal length, during which the system returns to an apparently, almost, normal condition. Each variety of the type observes its own law of periodicity, be it quotidian, tertian or quartan—a pathological mystery yet unsolved. Exposure to the morbific influence for a certain time, varying with different subjects, is necessary to the development of the disease. The period of incubation may be a few days only, or a few weeks, or it may be many months. Analogously, we find that a large proportion of drinkers are *periodical* drunkards. The intermission between paroxysms of debauch may not be as uniform in a given case as in those of intermittent fever, but they are uniform enough to plainly stamp them as "periodic." The more common drunkard is not he who drinks every day, but he who has a "spree" once in just about so long. And as a variable time, in different subjects, is required for the specific materies morbi to produce intermittent fever, so indulgence in drink varies in the time necessary to establish the alcoholic habit. In both classes of patients we find an approach in the system to a normal standard, but in both the disease is only temporarily quiescent. In intermittent fever auxiliary causes, as exposure to cold, over-exertion or excesses in eating or drinking are sometimes necessary to give efficiency to the special cause of disease, and in alcoholism the same causes may bring on the slumbering desire for drink. How often is a "bad cold," "a headache" or "fatigue" pleaded as the incentive to a debauch.

There is a second class of drinkers in whom the disease has assumed a more continued type, with remissions instead of intermissions; that is, the patient is impelled to drink more or less every day, with occasional periods of excessive debauch. We have in remittent fever the analogue of this type of alcoholism. Intermittent and remittent fevers are mutually convertible into each other, both are due to the same specific cause, and both succumb to the same specific remedies. In remittent, on the advent of the febrile stage, the movement continues for a variable length of time, from six to forty-eight hours, and then notably subsides; the skin becomes moist, the frequency of the pulse is reduced, but the fever does not entirely disappear; there is not complete apyrexia; there is a remission, but not an intermission. Sometimes the remission is marked, sometimes it is slight. After a time the febrile movement is renewed with as much or more intensity as at first. Nausea and vomiting are prominent symptoms. The matters vomited are yellowish or greenish in color, there is distress of the stomach and epigastric tenderness. The functions of the mind are often notably disturbed. Jaundice occurs in a certain proportion of cases. What a remarkable resemblance do we here find to that type of alcoholism seen in the steady drinker! As with the victim of remittent, no intermission occurs in which the physical system is free from the power of the disease, so with the steady drinker, no intermission gives relief to the disease-permeated body or beclouded intellect. To-day his perverted appetite calls for somewhat less of the customary poison, but to-morrow it demands more to make up the temporary loss. To make the analogy more complete and more surprising, distress of the stomach and epigastric tenderness are present, nausea and vomiting common, and jaundice not infrequent.

A graver type of malarial affections is pernicious fever, due to the same specific agent, but characterized by phenomena more remarkable and much more apt to end in death, than those of the milder types. It is, indeed, one of the most formidable affections with which the physician is called to deal. In some cases profound coma may be present; in others active delirium of a violent and dangerous character. The essential morbid conditions are the same as in the milder types but intensified and exaggerated. The materies morbi of the disease makes a most profound and alarming impression upon the powers of life. It is the last and most virulent exhibition of the malarial poison. Its onset may be similar to that of chills and fever, or remittent; in two or three days the skin becomes livid, shrunken and sometimes clammy, countenance anxious, thirst intense, stomach excessively irritable, mucous vomitings, pulse small, weak and rapid, restlessness marked, or in some cases, drowsiness and hesitation of speech; and coma or tetanic spasms may supervene.

If death do not take place in the collapse, partial or complete reaction follows in a few hours, but the next day at about the same hour another paroxysm occurs, more dangerous than the first, and if a third be allowed, it is generally fatal. In that condition known as delirium tremens we have the analogue of pernicious fever. The two conditions have many symptoms in common. The access of delirium tremens is marked by insomnia, anorexia, depression of spirits, and muscular tremor, notably of the tongue. In two or three days mental aberration of a peculiar character supervenes and remarkable restlessness. Illusions crowd the distempered brain. If a favorable termination happen, sleep at length ensues, out of which the patient awakens free from delirium. If the affection pursue an unfavorable course, the insomnia persists with great prostration, delirium continues, the body is bathed in perspiration, the pulse becomes more weak and frequent, and coma generally ends the scene.

Now, aside from those theoretical reasons for confidence in cinchona for the cure of alcoholism educed from the above analogies, I have undeniable and positive proofs of the cure of drunkenness by this agent. I have as much confidence in it as I have in the sulphate of quinia in intermittents. I do not judge by hearsay, but by actual experiment and observation. I know of radical, and, so far, lasting cures of confirmed drunkards. The effect of the medicine is not to create a disgust for alcohol, but a total indifference. If a patient will voluntarily recommence the use of alcoholics, his disease returns, just as the patient cured of intermittent will again contract that disease if again exposed to malarial poison. There are plenty of inebriates of naturally low and brutal instincts who will drink for the effects of it, whether they have a drunkard's craving for it or not. Is it not of this class that the "relapses" are made up? Have not the failures been due to inefficient administration of the medicine? My experience with the "cure" has been so entirely different from that of Dr. Earle that I would like to have physicians generally test the matter and report through the JOURNAL AND EXAMINER.

RICHMOND, ILL., February 25, 1880.

